# Attention deficits in Brazilian health care workers with chronic pain

**DOI:** 10.3389/fpsyg.2022.1024584

**Published:** 2022-10-24

**Authors:** Sergio L. Schmidt, Ingrid M. Araguez, Vithória V. Neves, Eelco van Duinkerken, Guilherme J. Schmidt, Julio C. Tolentino, Ana Lúcia T. Gjorup

**Affiliations:** ^1^Department of Neurology, Federal University of the State of Rio de Janeiro, Rio de Janeiro, Brazil; ^2^Department of Medical Psychology, Amsterdam University Medical Centers, Vrije Universiteit, Amsterdam, Netherlands; ^3^Amsterdam Diabetes Center/Department of Internal Medicine, Amsterdam University Medical Centers, Vrije Universiteit, Amsterdam, Netherlands

**Keywords:** chronic pain, neuropsychology, attention, continuous performance, cognition

## Abstract

The impact of COVID-19 on chronic pain (CP) in non-infected vulnerable South American subjects is unknown. Healthcare workers (HCWs) are at increased risk for CP. During the pandemic, many HCWs with CP kept working. Knowing how cognition is affected by CP in these subjects is an important subject for work safety. The attention domain has a pivotal role in cognition. Previously, the Continuous Visual Attention Test (CVAT) was applied to detect specific attention deficits in fibromyalgia patients. The present investigation described CP prevalence in non-infected Brazilian HCWs during the pandemic and assessed HCWs’ attentional performance with the aid of the CVAT. This study was carried out at a reference University Hospital in Rio de Janeiro, Brazil. HCWs of both sexes, aged 20 or older, were interviewed from August to December 2020. A 90-second version of the CVAT was performed. The average reaction time to correct responses and the respective intraindividual reaction time variability for correct responses to target (VRT) was determined. Omission and commission errors were also calculated. Then, for each participant we calculated the *Z*-scores of the CVAT variables based on the distribution of CVAT performance of 211 healthy subjects (reference-comparison group). HCWs with *Z*-scores > 1.64 were classified as significantly impaired. From the 154 selected HCWs, 72 reported CP during the pandemic (prevalence = 47%). Post hoc ANCOVAs showed that the average correct VRT was significantly higher in the CP group than in the non-CP group (*F* = 4.99, df = 1/150, *p* = 0.027, η^2^ = 0.032). The percentage of participants with impaired VRT performance was 30% (*n* = 21) in the CP group and 16% (*n* = 13) in the non-CP group. The difference between these two propositions reached significance (χ^2^ = 3.96, df = 1, *p* = 0.047). As VRT is associated with the sustained-attention subdomain, our data suggest that this subdomain is disrupted in the CP group.

## Introduction

The International Association for the Study of Pain defines chronic pain (CP) as an unpleasant sensory and emotional experience that persists for more than 3 months (Raja et al., 2020). Population estimates for CP prevalence varies by case definition, ascertainment methods, time, and place. Previous studies have shown that CP affects 13–40% of adults worldwide (Mills et al., 2019). In the US, a recent survey found that 50.2 million adults (20.5%) reported pain on most days or every day (Yong et al., 2022).

It has been proposed that CP conditions can be triggered by psychosocial stressors (McBeth et al., 2005; Generaal et al., 2016; Yavne et al., 2018). However, the incidence of pain complaints did not change among New York and New Jersey residents surveyed after the World Trade Center 9/11 attacks (Raphael et al., 2020). Similarly, catastrophic events, such as earthquakes, fires, or other naturally occurring catastrophic situations have not invariably led to an increase in chronic somatic symptoms (Pillow et al., 1996). These studies suggest that not all psychological stressors will trigger or exacerbate CP. In contrast, veterans showed combat-related psychiatric symptoms closely associated with CP (Xue et al., 2015). Therefore, simultaneous or over time exposure to many stressors can potentially increase the risk for future CP.

The COVID-19 pandemic has many characteristics that could increase the prevalence and incidence of CP. Importantly, the effects of the COVID-19 pandemic on CP could manifest in both infected and non-infected individuals due to routine life and sleep disruptions and physical, psychological, and social stressors which are all factors that can contribute to the chronic pain experience (Van Houdenhove, 2000; Meints and Edwards, 2018; Varallo et al., 2022). However, the impact of COVID-19 on CP is still a matter of controversy. Colloca et al. (2021) reported pain outcome improvement during the stay-at home period in participants not diagnosed with COVID-19. Mun et al. (2022) showed that individuals with chronic pain overall did not experience significant exacerbation of pain and opioid misuse during the COVID-19 pandemic. In contrast, another study in the US showed that approximately 25–30% of individuals reported exacerbation of pain severity and pain interference since the COVID-19 outbreak (Mun et al., 2021). Similarly, a research in Spain reported adverse effects of the COVID-19 pandemic on CP (Nieto et al., 2020). In accordance of the latter studies we hypothesize that the COVID-19 pandemic may cause an increase in new-onset CP among non-infected subjects.

If little is known about the effect of the COVID-19 pandemic on CP, even less is known about its impact on specific vulnerable populations. In general, healthcare workers (HCWs) are at increased risk for development of CP and constitute a vulnerable population (Barski et al., 2020). Individuals working in low- and middle-income countries are particularly disadvantaged due to limited resources and shortage of healthcare workers. During the pandemic, many HCWs kept working despite suffering from CP (Ozen and Cakmak, 2021). Therefore, we conducted a study to address this issue. We sought to provide a general exploratory perspective of the incidence and prevalence of CP among HCWs based on a cross-sectional face-to-face survey in one of the most affected South American countries.

As mentioned, HCWs are particularly vulnerable to CP during the current pandemic. An additional question that arises is how cognition is affected by CP in these subjects. This is important, as CP affecting cognition might lead to work-related errors. Interestingly, studies using functional MRI reported that CP patients have lower dorsolateral prefrontal cortex (DLPFC) and the posterior parietal cortex (PPC) connectivity when compared to controls (Apkarian et al., 2004; Baliki et al., 2008; Seminowicz et al., 2011; Pujol et al., 2014; Seminowicz and Moayedi, 2017). Attention is one of the core functions of the DLPFC and PPC. This was demonstrated by a study measuring regional FDG-PET metabolism during a visual attention test, the Continuous Visual Attention Test (CVAT) (Schmidt et al., 2008a). In a previous investigation, the CVAT was applied to assess attentional performance in fibromyalgia patients (Schmidt et al., 2008b), showing that CP patients had a poorer performance compared to non-CP subjects. This suggests that CP might affect HCWs’ attentional performance and the CVAT could be helpful to assess this.

This investigation sought to describe the prevalence of CP among non-infected Brazilian HCWs during the COVID-19 pandemic. Then, with the aid of the CVAT, we assessed the attentional performance of HCWs with CP and compared their average to those without CP. Finally, we applied categorial cutoffs to the two groups to verify whether the percentage of subjects with relevant impaired attention was higher in HCWs with CP. We expect that the timely recognition of attention deficits among workers with CP who are required to keep working would allow for more appropriate treatment to mitigate the potential impact of CP on work safety.

## Materials and methods

### Initial sample

The study was carried out among HCWs at the University Hospital of Federal University of Rio de Janeiro, located in the city of Rio de Janeiro, Brazil. Two hundred (200) HCWs, of both sexes, aged 20 or older, were interviewed from August to December 2020. The healthcare workers (HCWs) who worked in the hospital during this research period were invited to participate in the present study by email. The researchers informed the study aims and the procedure of data collection. After this invitation, those who agreed to participate were contacted by the research team.

A specifically developed questionnaire, completed by participants with their identification details (age/sex/race/education level), questioned whether they suffered from pain and, if so, for how long (duration/if appeared before or after the pandemic) have they felt such pain and where (head, lumbar, cervical, inferior or superior members).

The study was conducted in accordance with the Declaration of Helsinki, and all participants provided written informed consent. The local ethical review committee approved the research protocol (39365120.8.0000.5258).

### Inclusion and exclusion criteria

We included HCWs with pain complaints that had persisted for more than 3 months and that were not related to trauma, surgery, or cancer. We excluded participants who reported using any of the following drugs: antidepressants, benzodiazepines, modafinil, opioids, gabapentin, and pregabalin. We also excluded subjects with previous or current COVID-19 diagnosis, individuals with neurological or psychiatric conditions, and those who handed over incomplete questionnaires.

### Evaluation of attention performance

**FIGURE 1:**
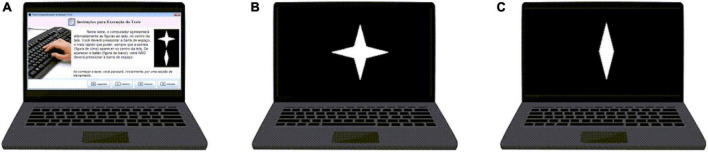
Continuous Visual Attention Test (CVAT). This version of the CVAT begins with written instructions on the screen **(A)** The computer alternately displays the indicated figures at the center of the screen. The participant must press the spacebar using her/his dominant hand as fast as she/he can whenever the star appears at the center of the screen. The participants are instructed to not press the spacebar if the other figure appears. These instructions are read aloud by the interviewer. The target **(B)** remains on screen for 250 milliseconds (ms). The non-target **(C)** also remains on screen for 250 ms. There was one block with 90 trials (two Figures – both targets and non-targets – were presented, one each time). The interstimulus interval was 1 s. The entire test took 1.5 min to be completed. Variables: Omission errors (OE), Commission errors (CE), Average reaction time for correct responses (RT), and reaction time variability (VRT). The CVAT is open for registered psychologists upon request. English, Portuguese, and Spanish versions are available.

The 90-second version of the CVAT was used. The CVAT is a computerized Go/No-Go test (Figure 1) for this study, and a fully description can be found in a previous publication (van Duinkerken et al., 2021). In brief, target and non-target stimuli were presented at the center of a screen for 250 milliseconds, with a 750- millisecond interstimulus interval. Of the 90 trials, 72 (80%) were targets, and 18 (20%) were non-targets.

Subjects were seated in front of the computer in such a way as to allow the dominant hand to be placed over the keyboard. The distance to the center of the monitor was of approximately 50 cm. Instructions were shown on the screen, then reinforced by the examiner. A practice session, in which no error could be made, was presented before testing commenced. In case of error, the practice session was automatically repeated.

The average reaction time for correct responses to target was calculated for each participant. Additionally, as reaction times vary throughout the test, intraindividual reaction time variability for all correct responses to target was determined. VRT was estimated by the standard deviation (SD) of each person’s responses calculated over all correct RT trials, because SD is considered the simplest and is the most used measure of VRT (Saville et al., 2011; Dykiert et al., 2012). In addition, several studies have reported that different variability metrics tended to produce similar results (Myerson et al., 2007; Saville et al., 2011; Dykiert et al., 2012). However, one problem that affects this metric is that some studies have described a relationship between mean RT and SD in a some neurological diseases (Wagenmakers and Brown, 2007; Tamm et al., 2012). To account for any possible association between mean RT and SD we also calculated the coefficient of variation, which is the ratio of VRT (i.e., SD) to the individual mean correct RT. Omission errors (no response to target) and commission errors (response to a non-target) were also calculated.

### Reference group

To determine CVAT cutoffs, we used previous data obtained from a large sample of healthy subjects (*n* = 211). These subjects completed the CVAT between June and December 2019. They all had normal neurological exams, absence of visual and hearing impairments, no psychiatric complaints, were not using psychiatric medication, and had a mini-mental status examination in the normal range.

### Statistical analysis

Demographic variables were analyzed using an independent sample *t*-test for normally distributed variables, a Kruskal–Wallis test for non-normally distributed variables, or a χ^2^ test for categorical variables.

First, a MANCOVA was performed including correct reaction time, correct reaction time variability, omission and commission errors as dependent variables, and presence of CP (yes or no) as independent variables. The same statistical procedures were also performed replacing VRT and RT with the respective coefficient of variability (VRT/RT). Age and sex were used as covariates. Box’s M test was used to assess the homogeneity of covariance matrices. In case of a significant overall MANCOVA, the post hoc ANCOVA of each dependent variable was checked for statistical significance. A significant MANCOVA suggests that at least one dependent variable is different between the groups, thus allowing for further post hoc testing. For MANCOVA and each one of the univariate ANCOVAs, η^2^ (Eta squared) was computed to calculate the effect size of results (Cohen, 1973). Cohen suggests that η^2^ = 0.01 should be considered a “small” effect size, 0.06 represents a “medium” effect size, and 0.14 a “large” effect size (Cohen, 1973).]. Corrections for multiple comparisons were performed with the Bonferroni method. A MANCOVA/ANCOVA approach was chosen as it has been shown to give robust results even when variables are not normally distributed (Olejnik and Algina, 1984). A *p* < 0.05 was considered statistically significant.

To investigate the number of HCWs with relevant attentional deficits, we calculated the participants’ *Z*-scores for each CVAT variable considering the means and the standard deviations (SD) of the reference group. A *Z*-score of 1.64 indicates that the performance is 1.64 SD to the right of the mean of the reference group, which represents a cumulative probability of 95%. In other words, a *Z*-score of 1.64 or higher indicates that performance is in the 5th percentile of the reference group. Therefore, for each CVAT variable, a participant’s *Z*-score greater than 1.64 was classified as being significantly impaired. Differences in the proportion of subjects with attentional impairments between HCWs with and without CP were tested with chi-square tests.

## Results

### Participants

From August to December 2020, 200 HCWs were interviewed and tested. After applying the exclusion criteria, 154 participants were included (a total of 46 participants – 7 associated with the use of medications – were excluded). Among these 154 subjects, 72 reported CP that lasted 3 months or more during the pandemic (57 participants reported CP starting prior to the pandemic and 15 CP starting during the pandemic). Demographic variables are shown in Table 1.

**TABLE 1:** Demographic variables.

	Control (*n* = 82)	CP starting before the pandemic (*n* = 57)	CP starting during the pandemic (*n* = 15)	Reference (*n* = 211)
**Age (years)** Mean ± SD	40.0 ± 12.0	43.3 ± 11.4	36.5 ± 11.7	38.2 ± 12.1
**Sex** Male Female	33 (40.2%) 49 (59.8%)	7 (12.3%) 50 (87.7%)	2 (13.3%) 13 (86.7%)	109 (51.7%) 102 (48.3%)
**Race self-referenced** Whites Nonwhites	42 (51.2%) 40 (48.8%)	21 (36.8%) 36 (63.2%)	6 (40.0%) 9 (60.0%)	N/A**
**Educational level** Primary school High school College or above	0 23 (28.0%) 59 (72.0%)	1 (1.8%) 10 (17.6%) 46 (80.6%)	0 4 (26.7%) 11 (73.3%)	42 (20.0%) 93 (44.0%) 76 (36.0%)
Pain location* Head Cervical Upper limbs Lumbar Lower limbs	–	12 (24.5%) 5 (10.2%) 4 (8.2%) 16 (32.6%) 12 (24.5%)	5 (33.3%) 1 (6.7%) 3 (20.0%) 4 (26.7%) 2 (13.3%)	–

CP: Chronic pain. *Eight subjects with CP starting before the pandemic did not inform pain location. **Not available.

### Prevalence and incidence

**FIGURE 2:**
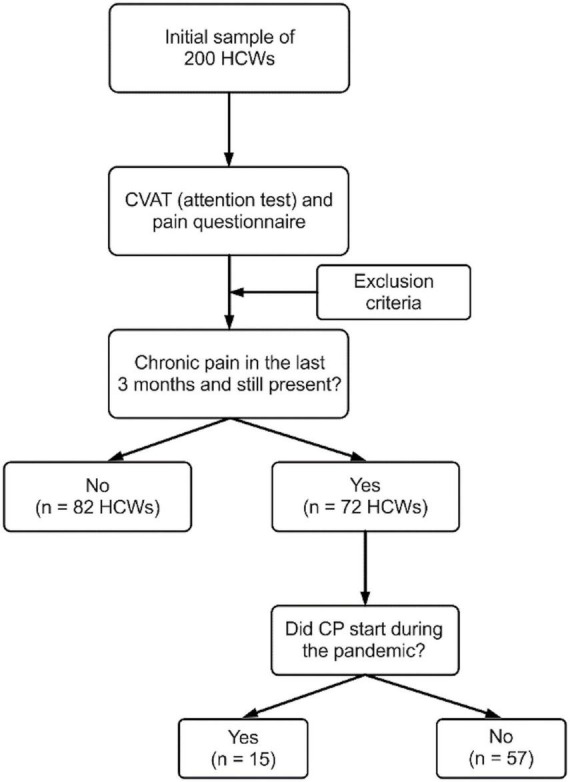
Flowchart of the study design. HCWs, healthcare workers; CVAT, Continuous Visual Attention Test; CP, chronic pain.

From the 154 participants, 72 reported CP during the pandemic – a 47% prevalence. As 57 subjects reported CP before the pandemic, 97 participants did not have CP before the pandemic. Among these 97 subjects, 15 reported CP starting during the COVID-19 pandemic. This gives a 15.4% incidence in 5 months (Figure 2). As 57 subjects had CP before the pandemic among the selected sample (*n* = 154), this gives a 37% prevalence before the pandemic.

### Mean group differences in attentional performance

**FIGURE 3:**
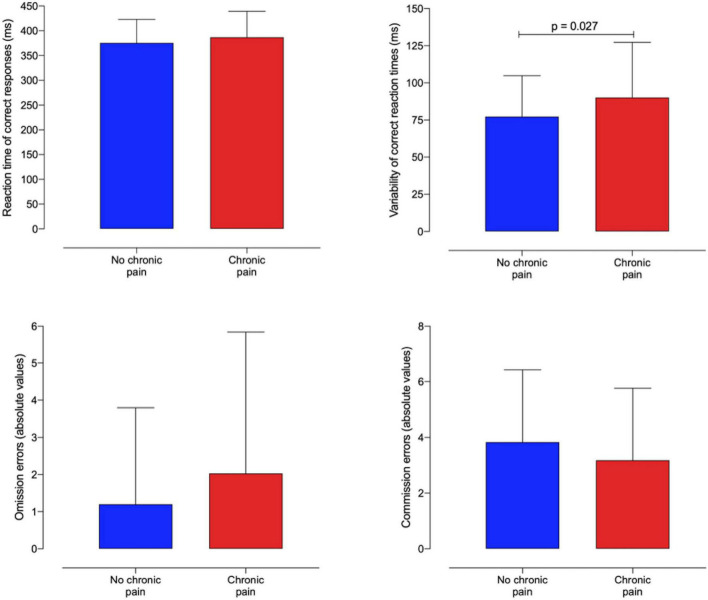
Continuous Visual Attention Test (CVAT) performance. Bar graphs displaying mean with standard deviation per group for each CVAT variable. Chronic pain (CP) patients present higher reaction time variability than non-CP participants.

Figure 3 shows the average reaction time, reaction time variability, and commission and omission errors dividing the participants into CP ad non-CP, based on presence of CP at the moment of the CVAT assessment. The overall MANCOVA reached statistical significance (*F* = 2.76, df: 4/147, *p* = 0.031, η^2^ = 0.07). The post hoc ANCOVAs showed that the average correct response time variability was, statistically speaking, significantly higher in the CP group than in the non-CP group (*F* = 4.99, df: 1/150, *p* = 0.027, η^2^ = 0.032). Although the average reaction time and the number of omission errors were also higher in the CP vs. non-CP group, none had statistical significance.

When the VRT and RT were replaced with the corresponding coefficient of variability (VRT/RT), the effect of CP on attention performance remained significant (MANCOVA, *F* = 3.03, df = 3/148, *p* = 0.031, η^2^ = 0.06). The post hoc ANCOVAs showed that the coefficient of variability was significantly higher in the CP group than in the non-CP group (*F* = 5.50, df: 1/150, *p* = 0.020, η^2^ = 0.035) whereas omission and commission errors did not reach significance.

### Number of healthcare workers with impaired attention

Based on the VRT distribution of the 211 subjects of the reference group, we determined the number of HCWs with *Z*-scores > 1.64 (i.e., subjects below the 5th percentile). Twenty-one participants (30%) with CP and 16% (*n* = 13) without CP had VRTs in the impaired range (*Z*-score > 1.64). The difference between these two proportions reached significance (χ^2^ = 3.96, df = 1, *P* = 0.047). We did not use Fisher’s Exact Test because none of the cell counts was less than 5. As the total *N* for the 2 × 2 chi-square table was greater than 40, the Yates continuity correction was not used.

## Discussion

We showed that CP prevalence increased among non-infected Brazilian HCWs during the COVID-19 pandemic. These HCWs kept working despite the presence of CP. Based on average VRT those with CP showed a significant deficit in the ability to sustain attention compared to HCWs without CP. The percentage of impaired VRT subjects in the CP group was significantly higher than that of the non-CP group.

A previous study estimated that CP prevalence reached 38% in Brazil (de Souza et al., 2017). The same study showed a 40% prevalence specifically in the Southeast region, including the city of Rio de Janeiro (de Souza et al., 2017). In line with these findings, we found a 37% CP prevalence before the current pandemic. When we considered the new cases during the pandemic, CP prevalence rose to 47%, i.e., higher than before the COVID-19 outbreak. In the current study, CP new cases during the pandemic reached 15% in 5 months (15 new cases), which is considerably higher than the incidence reported in other countries. However, these data should be interpreted with caution due to several limitations. First, the small sample size challenges while evaluating populational incidence and prevalence. Secondly, the same individuals used to calculate CP incidence were also present in estimating CP prevalence during the pandemic. However, for the first time, we described a preliminary perspective of CP prevalence during the COVID-19 outbreak in a South American country, despite these limitations. Following the hypotheses raised by Clauw et al.(Clauw et al., 2020), these preliminary data show that the COVID-19 pandemic increased CP prevalence among HCWs, probably because of high psychosocial stressors extending over many months, as experienced by these workers during the outbreak.

The high frequency of CP among HCWs who had to keep working during the COVID-19 pandemic raised a concern about their actual cognitive abilities. Here we found significant attentional deficits during the performance of the CVAT by HCWs with CP. Indeed, there are several studies on the relation between brain alterations and maladaptive cognitive and emotional factors in patients with CP, due to the presence of a cerebral plastic time-dependent reorganization related to the maintenance of pain (Malfliet et al., 2017). However, it should be mentioned that changes in plasticity are not the only mechanisms that can underlie these phenomena. Central sensitization has been described in CP and was found to be correlated with gray matter volume decrease in the DLPFC as well as changes in the PPC (Cagnie et al., 2014). Furthermore, functional MRI studies using a Go/No-Go attention task in healthy volunteers showed the activation of a network comprising the frontoparietal cognitive control regions (Bellgrove et al., 2004; MacDonald et al., 2006). Consistent with this idea, several studies have shown that persistent and long-term pain affects brain function in response to attention tasks (Baliki et al., 2008; Schmidt-Wilcke et al., 2014). In accordance with these studies, we showed that most of the HCWs with CP had significant changes in the variability of reaction times as measured with the aid of the CVAT.

Adding the coefficient of variability to the analysis allowed for the study of the VRT variable controlling for RT (Flehmig et al., 2007). The present study showed a significant difference in the coefficients of variability between CP and non-CP participants. Thus, this finding gives further support for the hypotheses that the increase in the VRT seen in the CP group is not explained by any putative increase in RT. Similarly, other studies have reported higher reaction time variability among older adults when compared to younger adults, even when group differences in response speed were statistically controlled (Hilborn et al., 2009).

Reaction time variability reaction time fluctuations during the CVAT. In this study, HWCs with CP had more significant response time fluctuations than those without CP. There is literature available to support the hypothesis that a greater VRT is observed in several neurological conditions, such as Alzheimer’s disease or mild cognitive impairment (Schmidt et al., 2020, 2021). In addition, VRT was found to predict progression of cognitive decline from healthy aging to mild cognitive impairment (Cherbuin et al., 2010; Haynes et al., 2017). Moreover, previous studies have demonstrated that higher reaction time variability is related to worse cognitive functioning in domains such as sustained-attention, memory, intelligence, and information processing speed (Hultsch et al., 2002; Epstein et al., 2011; Simões et al., 2017). Furthermore, VRT is associated with white matter integrity in healthy adults (Bellgrove et al., 2004; Bunce et al., 2013). It should be mentioned that the evidence that greater VRT is associated with central nervous system dysfunction is independent of any increase in RT (Hilborn et al., 2009). Taken together these studies support that HCWs with CP presenting higher VRTs may show worse cognitive functioning, even without increased errors or RTs. Therefore, we suggest that HCWs with CP may show significant cognitive disturbances that would interfere with workplace safety.

This study has some limitations. First, the sample size used to estimate prevalence and incidence of CP is small. Secondly, because of the small number of subjects (*n* = 15), we did not have enough statistical power to study HCWs that started to report CP during the pandemic separately. One limitation of the present study is that the test used here consisted of 18 non-targets. Previous studies have shown that both reaction time and variability of reaction time can be reliably measured by tests as short as 52 s with 20 items (Manuel et al., 2019). Therefore, the 90-s CVAT has sufficient targets (72) to reliably measure reaction time and variability of reaction time. Regarding accuracy, however, the maximum number of commission errors (18 non-targets) limits the possibility to capture accuracy alterations. A commonly used measure related to accuracy is d’, which represents the sum of the normalized commission and omission errors. To calculate this measure, it is imperative that there are sufficient targets and non-targets allowing for participants to make mistakes. Because of the lower number of targets and especially of non-targets in the Go/No-Go task used in this study, we were less capable of capturing accuracy. Future studies using Go/No-Go tasks of longer duration should consider the inclusion of measures derived from signal detection theory, such as d’ (Nestor et al., 1990; See et al., 1997; MacMillan and Creelman, 2005; Hautus et al., 2021).

A recent longitudinal study reported that deficits in executive functions could impair the use of appropriate pain coping strategies and facilitate the development of chronic pain (Giusti et al., 2020). Therefore, a new investigation should address the association between the presence of executive dysfunctions and CP. In addition, pain catastrophizing measurements should be investigated in a future study because pain catastrophizing is related to brain areas involved in pain attention and top-down pain inhibition (Chehadi et al., 2017). Whether the reaction time variability in CP subjects is related to a greater risk of work accidents needs to be determined in future studies. In this regard, the standard deviation of reaction time measured in a reaction test was found to be significantly correlated with safe driving indexes [e.g., (Altarabichia et al., 2020)].

The strength of this study is that a reaction-time task (CVAT) was able to identify sustained-attention deficits in HCWs with CP. The CVAT is quick (90 s), requires little training, involves minimal linguistic capabilities, provides cost-efficient diagnostics (open to licensed psychologists), and has clear guidelines and norms. It should be mentioned that a previous study demonstrated that attentional performance might help predict which patients with CP will respond to duloxetine treatment even before they can demonstrate subjective improvements in pain perception (Schmidt et al., 2008b).

In conclusion, we demonstrated that a great number of HCWs with CP have a profile of reaction time performance that fluctuates more, i.e., is less stable compared to their counterparts without CP. This study shows that a simple reaction-time Go/No-Go instrument could be used to detect specific attention deficits in HCWs with CP.

## Data availability statement

The original contributions presented in this study are included in the article/supplementary material, further inquiries can be directed to the corresponding author.

## Ethics statement

The studies involving human participants were reviewed and approved by Plataforma Brasil (39365120.8.0000.5258). The patients/participants provided their written informed consent to participate in this study.

## Author contributions

SS participated of design of the study, collected and analyzed the data, and wrote the manuscript. IA and VN participated of the conception of the study, collected data, and critically revised the manuscript. GS participated of the data analyses and critically revised the manuscript. ED, JT, and AG collected data, analyzed data, and critically revised the manuscript. All authors contributed to the article and approved the submitted version.
